# Supplementary figures and images for: Mechanisms underlying different onset patterns of focal seizures
Source: PLoS Comput Biol. 2017 May 4;13(5):e1005475. doi: 10.1371/journal.pcbi.1005475 (PMC5417416; doi:10.1371/journal.pcbi.1005475)

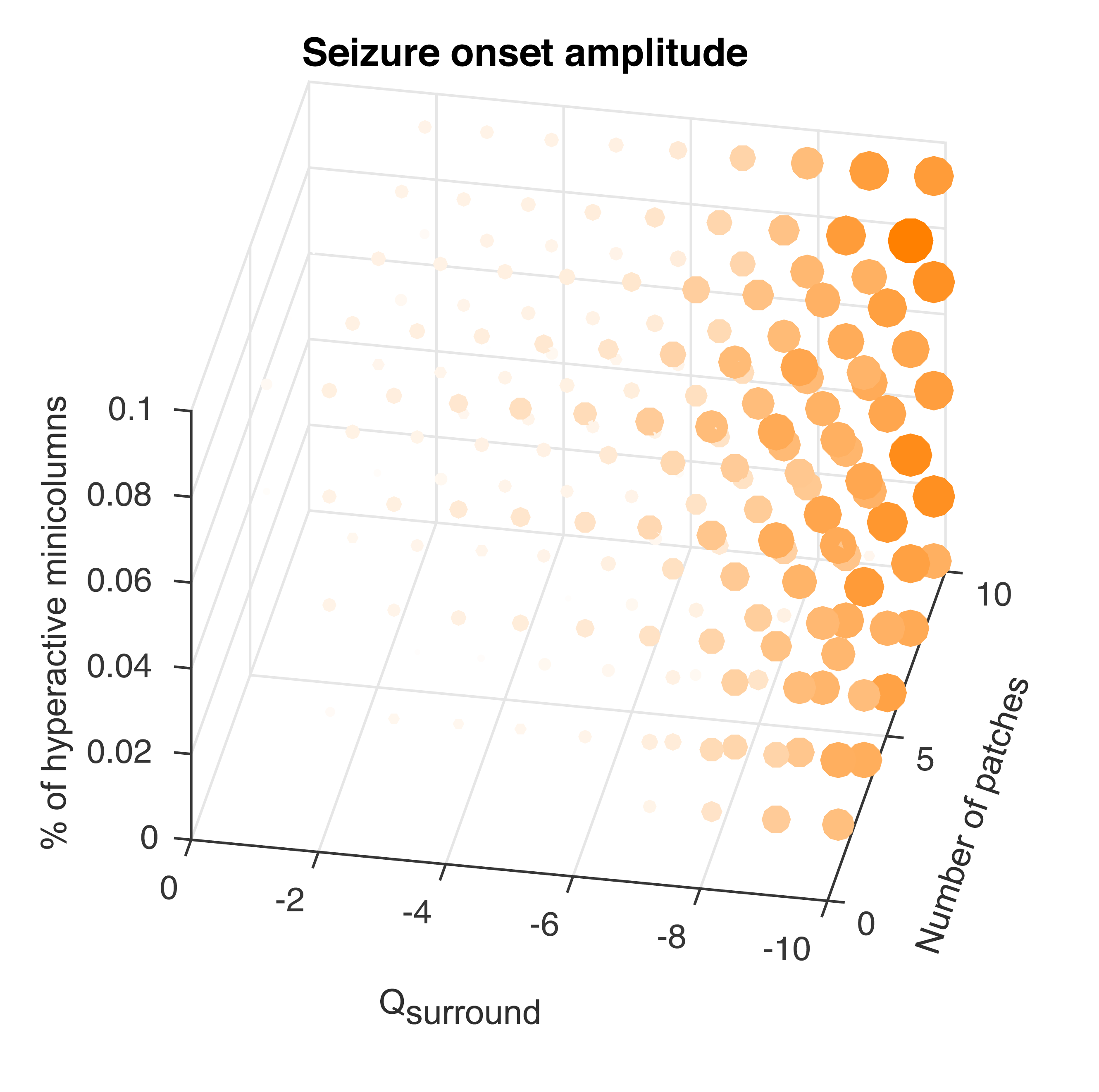

Supplement: S1 Fig — This is the equivalent figure to Fig 2 (a), only using Q as the parameter to change excitability. Seizure onset amplitude is, again, shown in the size of the marker. (PNG) [file pcbi.1005475.s001.png]
