# Supplementary material for: Mechanisms underlying different onset patterns of focal seizures
Source: PLoS Comput Biol. 2017 May 4;13(5):e1005475. doi: 10.1371/journal.pcbi.1005475 (PMC5417416; doi:10.1371/journal.pcbi.1005475)
Supplement: S1 Text — We show the bifurcation behaviour in the deterministic system, and specifically focus on the monostable state, and under what conditions microdomains can still recruit their surrounding tissue. (PDF) [file pcbi.1005475.s002.pdf]

# Text S2: Dynamics of the simulated cortical sheet

The treatment of the simulated cortical sheet in the main text is aimed at a broad audience and the results are presented in the clinical context of epileptic seizures. Here, we wish to treat the system in the domain of dynamical system, in order to provide the reader with more theoretical context. To this end, we first disentangle the deterministic behaviour of the system from the stochastic simulations. The system, as presented in current the paper (and our previous work [Wang et al., 2014]), can be treated as an ordinary differential equation (ODE) system, when the noise term $A * S(t)$ is removed:

$$\begin{aligned}\tau_E \cdot \frac{dE}{dt} &= -E + Sigm(C_{E\to E} \cdot E + C_{I\to E} \cdot I + P) \\ \tau_I \cdot \frac{dI}{dt} &= -I + Sigm(C_{E\to I} \cdot E + C_{I\to I} \cdot I + Q),\end{aligned} \tag{1}$$

All notations remain the same as in the main text. Note that we introduced equation 1 as the Wilson-Cowan system (two variable) for a single minicolumn, but $E, I, P, Q$ and $\tau$ can be understood as vectors, and the connectivity parameters $C$ as matrices, which then fully describe the model cortical sheet.

When simulating the system deterministically, we find the same dominant dynamics. The parameter space is also still preserved in terms of the monostable background region, the monostable oscillatory (seizure) region, and the bistable region. Fig. 1A shows a bifurcation scan in parameter $P$ and highlights these three parameter regions. We also note that the background state is a stable node, the seizure state is a stable limit cycle. The dominant frequency of the limit cycle is shown in Fig. 1B, and it changes between about 8 and 14 Hz. The bistability is also shown in Fig. 1C, where $P = -1$. We start of the system in the fixed point, and upon perturbation it transitions to the oscillatory state.

The description and analysis of these states in neural field system has a long history (see [Amari, 1977] for an example). The bistability between a fixed point and an oscillatory state in such systems is also described analytically and numerically in related systems [Kim et al., 2009], and they are also termed "bistable medium" (e.g. see [Bressloff, 2012]). In our model, we wish to point out that the dynamics do not arise from weakly coupled units, that have the necessary bifurcation structures already. In our case, the single unit does not transition to an oscillatory state when changing the input parameters (see Fig. S2 & S3 in [Wang et al., 2014]). The oscillatory state in our model actually arises due to the coupling to the neighbouring units.

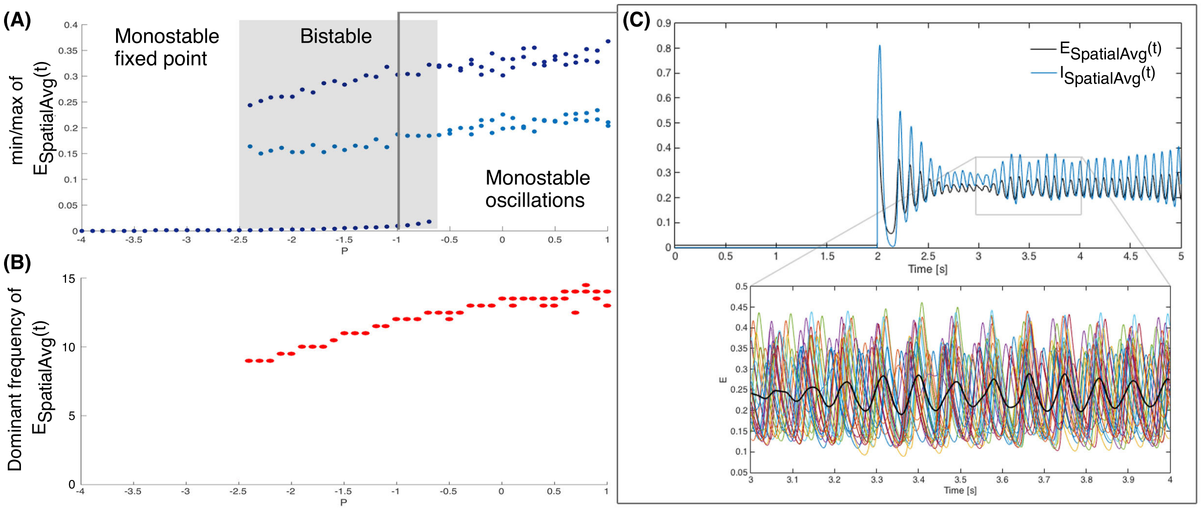


**Figure 1. Stable states for different values of $P$. (A)** Bifurcation scan of the stable attractors. Minima (light blue) and maxima (dark blue) of the time series $E_{SpatialAvg}(t)$ are shown at corresponding value of $P$. The scan is performed by first incrementing the value of $P$, and using the states of the last value of $P$ as the new initial conditions (slowly tracing the change in the attractor for different $P$). Then we also perform this by decreasing $P$ slowly to detect bistabilities. **(B)** The dominant frequency of $E_{SpatialAvg}(t)$ is shown for different values of $P$, where an oscillation is detected. **(C)** $E_{SpatialAvg}(t)$ and $I_{SpatialAvg}(t)$ are shown for $P = -1$. At time 2s, we perturb the system to induce a transition to the bistable oscillatory state. In the zoom-in the thick black line is $E_{SpatialAvg}(t)$, and the coloured lines show some example time series from a few minicolumns.

The second dynamic regime our paper is concerned with is the monostable background state. This is a stable node in our system, which when perturbed by a short transient activation simply returns back to the node. However, the question in this regime is whether (and under what circumstances) oscillatory activity can still be spread throughout the entire system. The inspiration in this case comes from the clinical literature reporting small pockets of localised seizure activity (microseizures), which seem to be able to occasionally recruit/penetrate into the surrounding tissue [Stead et al., 2010]. Hence, we decide to investigate the effect of persistent oscillatory input in the monostable background state. To achieve this, we introduced microdomains that are able to generate autonomous oscillatory seizure activity (by setting their $P$ to a higher value) in the main manuscript. We demonstrated that these microdomains can indeed recruit/penetrate into the surrounding tissue that is in the monostable background state.

Here, we wish to highlight some of the interesting theoretical aspects of this recruitment/penetration effect. To simplify matters, we introduce microdomains in the tissue that are sinusoidal oscillators (i.e. the minicolumns in the microdomains are being replaced by a sinusoid generator). This has the advantage

that we can control the oscillation parameters such as amplitude and frequency exactly, to study their effect. The arrangement of the microdomains can be seen in (Fig. 2A). First, we demonstrate that the recruitment/penetration effect is very much dependent on the value of $P_{surround}$ (Fig. 2B). In other words, in the monostable background state, it still matters what level of baseline input level is used, as to if it can be recruited/penetrated by oscillatory activity. This is also true for other parameters, such as $Q$, or $C_{I->E}$ (Fig. 2C,D). Indeed the proximity to the bistable state (red lines in Fig. 2B,C,D) appears to facilitate the full recruitment/penetration. This is also true for the case with noise input (more details can be found in our previous work [Wang et al., 2014], e.g. in Fig. 7. In this particular example, we used a stimulation frequency of 12 Hz, and the oscillation ranged between 0 and 1 (i.e. the sinusoid generator took the form of $sin(2\pi * 12 * t) + 0.5$).

To finish our investigation, we show that the stimulation frequency, and the spatial arrangement of the microdomains also matter in terms of recruitment (Fig. 3). $P$ was kept at $P = -2.5$ for this part of the investigation. The aspect of the spatial arrangement is essentially echoing Fig. 2 of the main manuscript. The frequency dependency is interesting, as it appears that an 8 Hz oscillation would not be able to achieve full recruitment, but an oscillation at or over 12 Hz would be, despite the intrinsic frequency of the oscillation being near 8 Hz (Fig. 1B). Similar frequency dependent observations have been reported before in one-dimensional media [Baier and Mller, 2004], and reaction-diffusion systems [Vanag and Epstein, 2006]. The intrinsic frequency of microdomains, when using the Wilson-Cowan minicolumn units with an increased $P$ (as in the main manuscript) is near 12 Hz. Hence full recruitment is observed in the main manuscript.

In conclusion, the dynamics we presented in our main manuscript can largely be categorised as either a bistability between a fixed point and a oscillatory state (high amplitude onset pattern), or a monostable fixed point that under the right oscillatory inputs and given the right internal parameter setting can still maintain a fully oscillatory rhythm (low amplitude onset pattern). Here we showed that these dynamics are found in the deterministic simulations as well, meaning that our observations in the main manuscript are mainly driven by the deterministic dynamics. We further demonstrated that there are many parameters and factors that can influence the recruitment process in the monostable deterministic case (parameter settings of the surround, spatial organisation of the microdomains, and oscillation frequency of the microdomains). This should give the reader a better overview of the deterministic foundations of the results presented in the main manuscript.

The observations made here might be amenable to analytical approaches, if one assumes a perfectly homogeneous connectivity (i.e. going to the neural field continuum approach, and removing the patchy remote connections). We did not attempt this here as we wanted to keep the nature of the connectivity in the form as used in the main manuscript to make the results comparable. Another approach of analysis that might also prove to be informative is to analyse a single minicolumn unit in terms of the input it gets over time, and the connectivity structure underlying the input. With such an analysis, it might be possible to delineate the properties of input (frequency, magnitude, amount) that is required for recruitment.

Finally, as an outlook, these suggested types of analysis might also lead to insight regarding how to control the oscillatory state (seizure state) in such tissues. For example, it has been suggested that spiral wave dynamics can be controlled by weak external forcing [Steinbock et al., 1993]. Under the right condition, it has also been shown that perturbations can break up pulse propagations through excitable media [Hagberg and Meron, 1998]. If seizures are to be understood as such oscillatory states in the tissue (supported by e.g. [Schevon et al., 2012]), and demonstrate dynamics such as spiral waves (see e.g. [Viventi et al., 2011] for evidence) as seen on continua, then models such as ours may serve as a useful tool to investigate questions regarding how to best control such oscillatory seizure states.

# References

Amari, 1977. Amari, S. (1977). Dynamics of pattern formation in lateral-inhibition type neural fields. *Biological cybernetics*, 27(2):77–87.

Baier and Mller, 2004. Baier, G. and Mller, M. (2004). Frequency-selective induction of excitation waves near sub-and supercritical Hopf bifurcation. *Physics Letters A*, 330(5):350–357.

Bressloff, 2012. Bressloff, P. C. (2012). Spatiotemporal dynamics of continuum neural fields. *J. Phys. A: Math. Theor.*, 45(3):033001.

Hagberg and Meron, 1998. Hagberg, A. and Meron, E. (1998). Propagation failure in excitable media. *Phys. Rev. E*, 57(1):299–303.

Kim et al., 2009. Kim, J. W., Roberts, J. A., and Robinson, P. A. (2009). Dynamics of epileptic seizures: evolution, spreading, and suppression. *J Theor Biol*, 257(4):527–32.

Schevon et al., 2012. Schevon, C. A., Weiss, S. A., McKhann, G., Goodman, R. R., Yuste, R., Emerson, R. G., and Trevelyan, A. J. (2012). Evidence of an inhibitory restraint of seizure activity in humans. *Nature Communications*, 3:1060.

Stead et al., 2010. Stead, M., Bower, M., Brinkmann, B. H., Lee, K., Marsh, W. R., Meyer, F. B., Litt, B., Van Gompel, J., and Worrell, G. A. (2010). Microseizures and the spatiotemporal scales of human partial epilepsy. *Brain*, 133(9):2789–2797.

Steinbock et al., 1993. Steinbock, O., Zykov, V., and Mller, S. C. (1993). Control of spiral-wave dynamics in active media by periodic modulation of excitability. *Nature*, 366(6453):322–324.

Vanag and Epstein, 2006. Vanag, V. K. and Epstein, I. R. (2006). Resonance-induced oscillons in a reaction-diffusion system. *Phys. Rev. E*, 73(1):016201.

Viventi et al., 2011. Viventi, J., Kim, D.-H., Vigeland, L., Frechette, E. S., Blanco, J. A., Kim, Y.-S., Avrin, A. E., Tiruvadi, V. R., Hwang, S.-W., Vanleer, A. C., Wulsin, D. F., Davis, K., Gelber, C. E., Palmer, L., Van der Spiegel, J., Wu, J., Xiao, J., Huang, Y., Contreras, D., Rogers, J. A., and Litt, B. (2011). Flexible, foldable, actively multiplexed, high-density electrode array for mapping brain activity in vivo. *Nature Neuroscience*, 14(12):1599–1605.

Wang et al., 2014. Wang, Y., Goodfellow, M., Taylor, P. N., and Baier, G. (2014). Dynamic Mechanisms of Neocortical Focal Seizure Onset. *PLoS Comput Biol*, 10(8):e1003787.

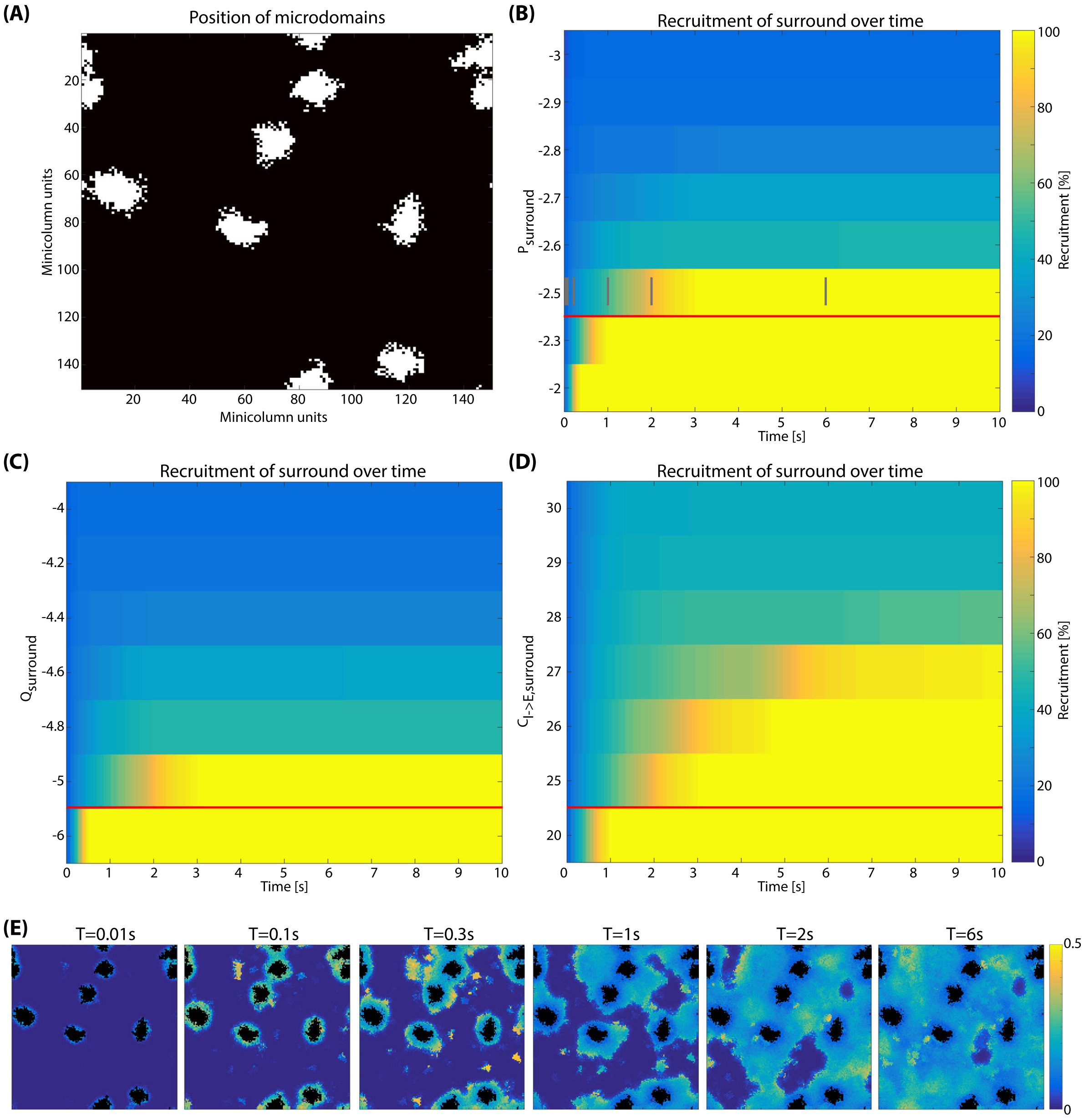


**Figure 2. Influence of the parameters of the surround on recruitment. (A)** Spatial position of microdomains (white) on the cortical sheet. This is the equivalent of 7.5% of the cortical sheet. Each microdomain minicolumn was replaced by a sinusoid generator $sin(2\pi * 12 * t) + 0.5$ for B, C, and D. **(B)** The percentage of recruitment of the surround is shown over time for different values of $P$. The red line indicates the bifurcation to the bistable regime. Note the recruitment is much quicker in the bistable regime ($P = -2.3$ and $P = -2$) than in the monostable regime, but does also slow down nearer the bifurcation threshold. The grey lines indicate the positions where we show the snapshots in E. **(C)** The percentage of recruitment of the surround is shown over time for different values of $Q$. The red line indicates the bifurcation to the bistable regime ($Q = -6$ is bistable). **(D)** The percentage of recruitment of the surround is shown over time for different values of $CI- > E$. The red line indicates the bifurcation to the bistable regime ($CI- > E = 20$ is bistable). (E) Snapshots of the cortical sheet activity in $E$ for different time points (indicated by grey lines in B). Black indicates the position of the microdomains.

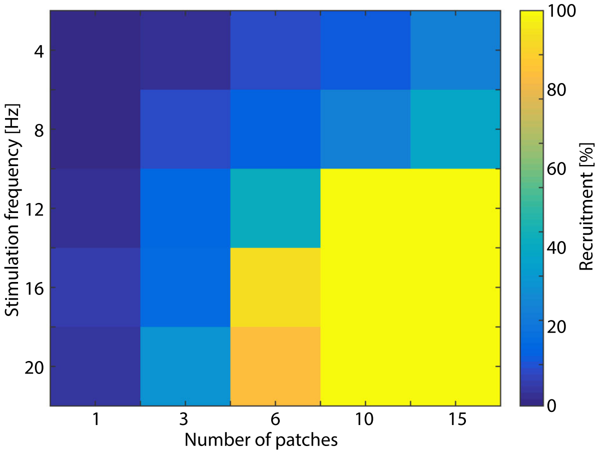


**Figure 3. Influence of stimulation frequency and microdomain arrangement in recruitment.** Depending on the microdomain arrangement and oscillation frequency, full recruitment may be able. For this scan, the total nuber of minicolumns in the microdomain was kept constant at 7.5% of the cortical sheet. For each value of number of subclusters, we generated five different random subcluster locations, and each scan point shows the average recruitment of the five cases. Here, recruitment is measured as the number of recruited minicolumns after 10 seconds of simulation. $P = -2.5$ for this scan.
